# Bacterial translocation aggravates CCl_4_-induced liver cirrhosis by regulating CD4^+^ T cells in rats

**DOI:** 10.1038/srep40516

**Published:** 2017-01-30

**Authors:** Haiyan Shi, Longxian Lv, Hongcui Cao, Haifeng Lu, Ning Zhou, Jiezuan Yang, Haiyin Jiang, Huihui Dong, Xinjun Hu, Wei Yu, Xiawei Jiang, Beiwen Zheng, Lanjuan Li

**Affiliations:** 1 The Collaborative Innovation Center for Diagnosis and Treatment of Infectious Diseases, Zhejiang University, Hangzhou, Zhejiang 310003, China; 2 The State Key Laboratory for Diagnosis and Treatment of Infectious Disease, the First Affiliated Hospital, College of Medicine, Zhejiang University, Hangzhou, Zhejiang 310003, China

## Abstract

Bacterial translocation (BT) is thought to play an important role in the development of liver cirrhosis, but the mechanisms have not been fully explored. This study aims to investigate the distribution of Treg (CD3^+^CD4^+^CD25^+^Foxp3^+^), Th17 (CD3^+^CD4^+^IL-17^+^), and Th1 (CD3^+^CD4^+^IFN-γ^+^) cells in the intestinal lamina propria, liver and blood and to explore their relationships with BT. Cirrhotic rats with ascites were induced by CCl_4_. We found that there were lower levels of total protein and albumin, lower albumin/globulin ratio, lower body weight and higher spleen weight and ascites volume in cirrhotic rats with than without BT. We found that BT may cause increase of Treg cells in the proximal small intestine and decrease of Th17 cells in the whole intestine and blood in cirrhotic rats. It may also aggravate the CCl_4_-induced decrease in Th1 cells in the whole intestine, liver, caecum, and blood and the CCl_4_-induced increase in Th17 cells in the liver and Tregs in the distal small intestine, colon, and liver. Our data suggest that BT may aggravate liver injury and decrease liver function via an interaction with CD4^+^ T Cells. The results of this study may be helpful for the development of new treatments for liver cirrhosis.

Bacterial translocation (BT) is a phenomenon in which intestinal bacteria or their products cross the intestinal barriers and enter the mesenteric lymph nodes (MLNs) and/or other extraintestinal organs^1^,^2^. An increasing amount of evidence indicates that BT is intimately associated with the development of liver cirrhosis and its complications^3^,^4^, such as hepatic encephalopathy, hepatopulmonary syndrome, hepatorenal syndrome, and liver failure^5^,^6^. Indeed, BT-associated infections and spontaneous peritonitis are major causes of death in patients with liver cirrhosis. Although bacterial overgrowth into the small intestine, intestinal mucosal barrier damage, and increased intestinal permeability have been observed in patients with liver cirrhosis with BT, the mechanism of interaction between BT and liver cirrhosis had not previously been fully explained^2^,^7^.

Several types of intestinal CD4^+^ T cells are critical to host defences against BT^8^. Th17 cells are distributed primarily in the intestinal lamina propria, especially in the small intestine, and are important for maintaining the integrity of the intestinal mucosal barrier and therefore crucial for preventing BT^9^,^10^,^11^. Treg cells accumulate in the intestine, where they play key roles in gut homeostasis and thereby affect BT^9^,^12^,^13^. For example, when Tregs are depleted, it can lead to an abnormal expansion of CD4^+^ T cells, resulting in intestinal inflammation^14^. Conversely, when Tregs are enriched, they can suppress other types of T cells, including Th17 and Th1 cells^14^, and this may also disrupt gut homeostasis. In liver cirrhosis, some gut disorders have been associated with BT and CD4^+^ T cells; these include overgrown bacteria in the small intestine, a damaged gastrointestinal barrier, increased intestinal permeability^4^,^9^, and an altered gut microbiome^10^,^11^,^12^. However, changes in intestinal CD4^+^ T cells and their interactions with BT have not previously been explored in liver cirrhosis.

In this study, we aimed to investigate differences in the enrichment of CD4^+^ T cells in the liver, blood and intestines between CCl_4_-induced cirrhotic rats with and without BT and to explore the relationship and the mechanism of interaction between BT and alterations in CD4^+^ T cells in liver cirrhosis.

## Results

### CCl_4_-induced cirrhosis increases the incidence of BT

**Figure 1:**
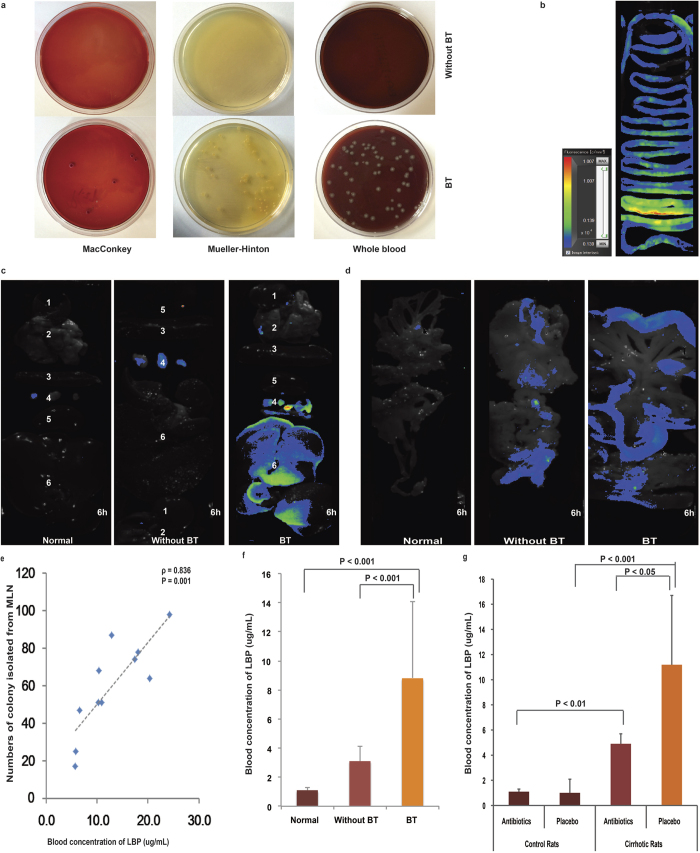
CCl_4_-induced cirrhosis increases the incidence of BT in rats. (**a**) Results of representative culture experiments using MLNs in cirrhotic rats with or without BT. (**b**) Correlations between the numbers of colonies isolated from the MLNs and plasma LBP levels were determined using Spearman’s rank test. (**c**) Plasma LBP concentrations in cirrhotic rats with and without BT and normal rats. (**d**) Plasma LBP concentration in antibiotic- or placebo-treated cirrhotic rats and normal rats. (**e**–**g**) A separate experiment was performed in which cirrhotic rats with ascites were administered 10^8^ RFP-tagged *E. coli* via gavage. Six hours later, RFP-marked *E. coli* were observed along the intestinal tract (**e**), in MLNs and the liver (**f**), and in the mesentery (**g**).

To determine whether cirrhosis affects BT, MLNs were isolated using sterile procedures for bacterial cultures according to the BT diagnostic criteria^8^. As shown in Fig. 1a, the MLNs obtained from 11/23 (47.8%) of the cirrhotic rats produced bacterial isolates in MacConkey, Mueller–Hinton and whole blood agar plates (herein defined as “BT” rats). However, no bacteria were isolated from the 12 normal rats. These results indicate that CCl_4_-induced cirrhosis increased the incidence of BT.

In addition to the number of bacteria obtained from MLN tissue cultures, we also used LBP, a soluble acute-phase protein that binds to bacterial lipopolysaccharide and elicits immune responses by presenting lipopolysaccharide to cell surface pattern recognition receptors, as an indicator of BT severity^15^,^16^,^17^. As shown in Fig. 1b, the plasma concentration of LBP was correlated with colony number, with a correlation coefficient as high as 0.918 (*P* < 0.001). Figure 1c demonstrates that the concentration of LBP was significantly higher in cirrhotic rats with BT than in normal control rats or cirrhotic rats without BT. Furthermore, the concentration of LBP was significantly lower in antibiotic- than placebo-treated cirrhotic rats (4.9 ± 0.8 versus 11.2 ± 5.5; P < 0.05) (Fig. 1d).

To further affirm that BT can result from intestinal bacteria in cirrhosis, 10^8^ RFP-marked *E. coli* were administered via gavage to normal and cirrhotic rats, and their organs were then examined using Clairvivo OPT Plus microscopy to track *E. coli* migration. Two hours later, a fluorescent signal was observed in the whole intestinal tract (Fig. 1e), and after 2 and 6 hours, 60% (6/10) of the cirrhotic rats with BT exhibited strong fluorescence in the MLNs and liver (Fig. 1f). In the lungs, the strongest signal was observed at 6 hours after lavage (Fig. 1f). In the mesentery, the fluorescence signal was strong in the cirrhotic rats with BT but weak in the normal rats (Fig. 1g). The data reveal that the marked bacteria translocated from the gut to the MLNs and into liver, demonstrating a possible pathophysiological route for the development of infectious complication in advanced cirrhosis.

### Cirrhotic rats with BT have worse liver functions, lower body weight, and higher spleen weight and ascites volume than those without BT

**Figure 2:**
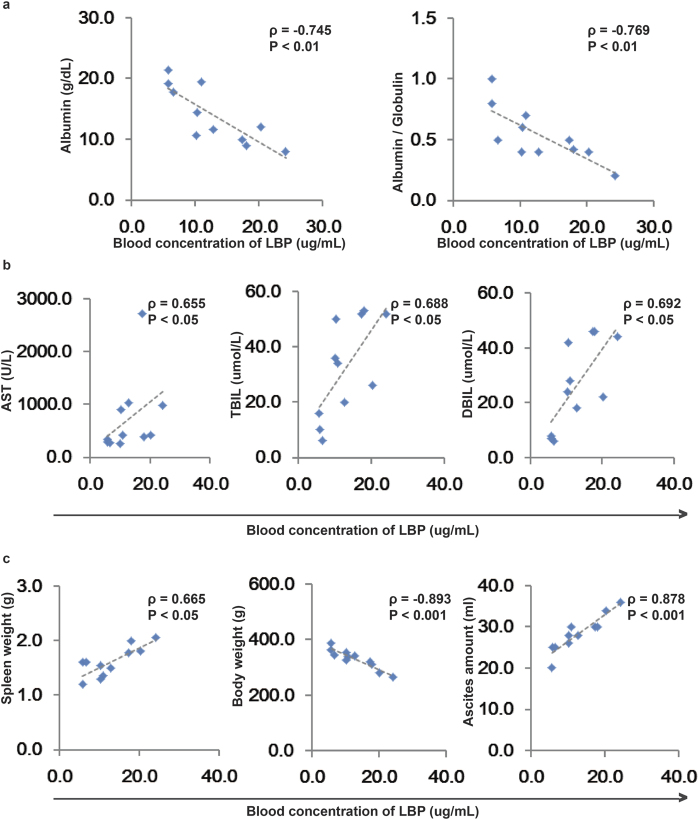
BT is associated with liver damage. Correlations between plasma LBP concentrations and albumin concentration and the albumin/globulin ratio (**a**), AST, TBil, and DBil levels (**b**), and spleen weight, body weight and the amount of ascites (**c**) in cirrhotic rats with BT. Spearman’s rank test was used.

As shown in Table 1, the serum levels of Globulin, ALP, GGT, TBil and DBil were significantly higher in cirrhotic rats with or without BT than in normal rats. The serum levels of ALT, AST and IBil were higher only in cirrhotic rats with BT, but were not significantly different between cirrhotic rats with and without BT. However, the serum levels of total protein and albumin and the albumin/globulin ratio was significantly lower in cirrhotic rats with BT than in normal control rats and cirrhotic rats without BT. The concentration of LBP was negatively associated with the albumin concentration (ρ = −0.745; P < 0.01) and the albumin/globulin ratio (ρ = −0.769, P < 0.01) (Fig. 2a) but was positively associated with the levels of AST (ρ = 0.655, P < 0.05), TBil (ρ = 0.688, P < 0.05), and DBil 0.692 (P < 0.05) (Fig. 2b). Serum levels of albumin and the albumin/globulin ratio were significantly higher and serum levels of ALT, AST, TBil and DBil were significantly lower in the antibiotic-treated cirrhotic rats than in the placebo-treated cirrhotic rats (Table 2). These results indicate that BT at least promotes an increase in serum AST levels and a decrease in serum albumin levels during the processes underlying CCl_4_-induced cirrhosis.

As shown in Table 1, there were lower final body weights, higher spleen weights and larger ascites volumes in the cirrhotic rats with BT than in the normal rats and/or cirrhotic rats without BT. The concentration of LBP was negatively associated with body weight (ρ = −0.893, P < 0.001) but positively associated with spleen weight (ρ = 0.665; P < 0.05) and the amount of ascites (ρ = 0.878, P < 0.001) (Fig. 2c). Final body weight was significantly higher, while spleen weight and ascites volume were significantly lower, in the antibiotic- than in the placebo-treated cirrhotic rats (Table 2). These results indicate that BT plays important roles in changing these factors.

### BT may increase Tregs in the proximal small intestine and decrease Th17 in the whole intestine and blood may in cirrhotic rats

**Figure 3:**
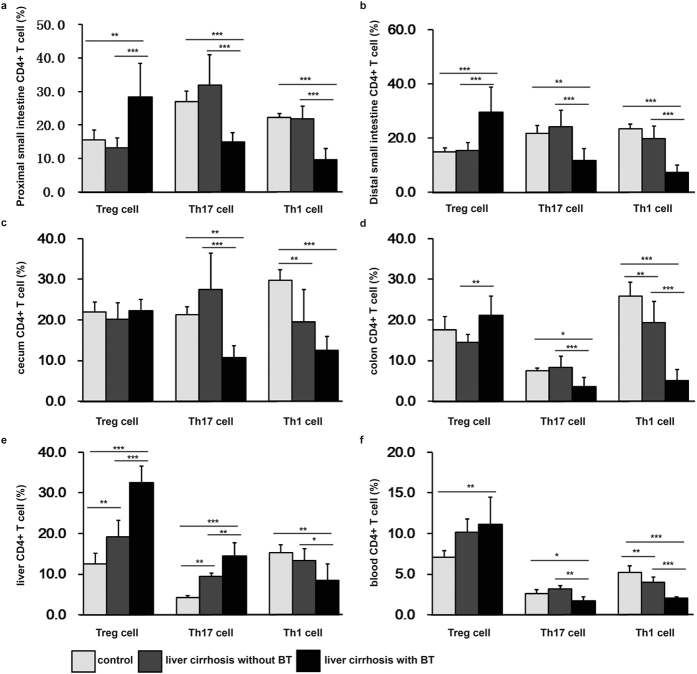
The percentages of Treg, Th17, and Th1 cells in cirrhotic rats without and with BT. Cells were isolated from the intestinal lamina propria, liver and blood. Cells were gated using the live lymphocyte population. The percentages of Treg (CD3^+^CD4^+^CD25^+^Foxp3^+^), Th17 (CD3^+^CD4^+^IL-17^+^), and Th1 (CD3^+^CD4^+^IFN-γ^+^) cells in the CD4^+^ T cell population were determined in the proximal and distal small intestine, caecum, colon, liver, and blood in the control group (n = 12), the liver cirrhosis without BT group (n = 12), and the liver cirrhosis with BT group (n = 11) using flow cytometry. The data are presented as the means ± SD. *P < 0.05, **P < 0.01, ***P < 0.001, one-way ANOVA.

Some of the changes in the percentage of different types of CD4^+^ T cells in some tissues appear to be associated with BT in liver cirrhotic rats because they were observed in liver cirrhotic rats both with BT and without BT but were not observed in normal rats or cirrhotic rats without BT. These changes included an increase in the percentage of Tregs in the proximal small intestine (Fig. 3a) and a decrease in the percentage of Th17 cells in the proximal and distal small intestine (Fig. 3a and b), caecum (Fig. 3c), colon (Fig. 3d) and blood (Fig. 3f). One possible explanation for this result is that BT cells may specifically induce the development or differentiation of only some types of CD4^+^ T cells, and another is that specific CCl_4_-induced alterations in the relative abundance of some CD4^+^ T cells lead to BT.

**Figure 4:**
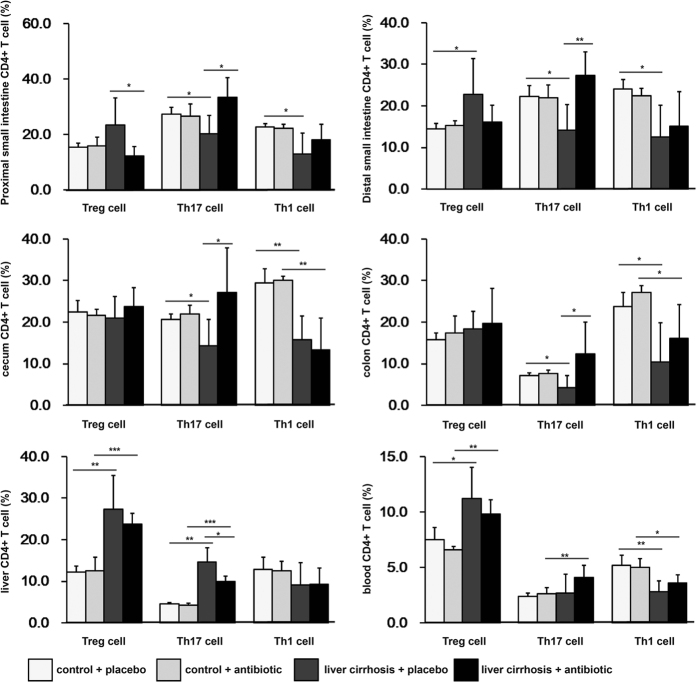
The percentages of Treg, Th17, and Th1 cells in antibiotic- and placebo-treated cirrhotic and normal rats. Cells were isolated from the intestinal lamina propria, liver and blood. Cells were gated using the live lymphocyte population. The percentages of Treg (CD3^+^CD4^+^CD25^+^Foxp3^+^), Th17 (CD3^+^CD4^+^IL-17^+^), and Th1 (CD3^+^CD4^+^IFN-γ^+^) cells in the CD4^+^ T cell population were determined in the proximal and distal small intestine, caecum, colon, liver, and blood of the antibiotic-treated controls (n = 8), the placebo-treated controls (n = 8), the antibiotic-treated liver cirrhosis group (n = 10), and the placebo-treated liver cirrhosis group (n = 10) using flow cytometry. The data are presented as the means ± SD. *P < 0.05, **P < 0.01, ***P < 0.001. Unpaired Student’s t test with Bonferroni’s correction was used.

To explore the cause and effect relationship between BT and the changes in the proportions of CD4^+^ T cells that occurred specifically in BT rats, as described above, we investigated the changes that occurred in these cells in rats with liver cirrhosis that were prevented from developing BT by intestinal decontamination, which was ensured using cocktails of antibiotics. As shown in Fig. 1g, gut decontamination resulted in a significantly (P < 0.05) lower concentration of LBP in the antibiotic-treated than in the placebo-treated cirrhotic rats. The percentage of Treg cells in the CD4^+^ T cell population in the proximal small intestine was lower (Fig. 4a), whereas the proportion of Th17 cells in the proximal and distal small intestine (Fig. 4a and b), caecum (Fig. 4c), colon (Fig. 4d) and blood (Fig. 4f) was higher in the cirrhotic rats with antibiotics than in the cirrhotic rats without antibiotics. However, the percentage of these cells was similar between cirrhotic rats with antibiotics and normal rats with either placebo or antibiotics. These data indicate that BT caused the changes observed in these cells.

**Figure 5:**
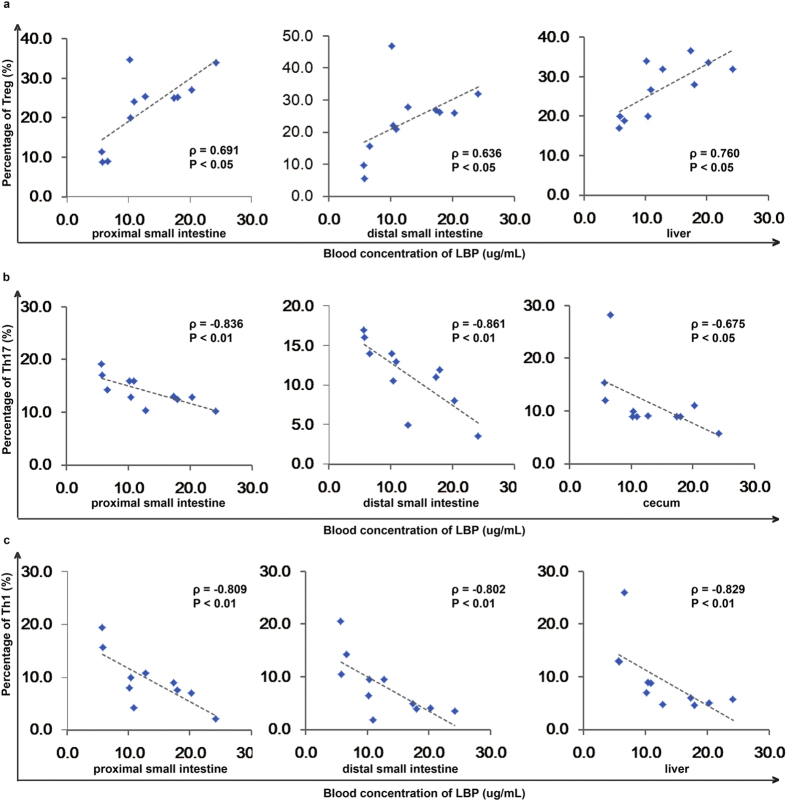
BT is associated with changes in the cellular immunity response. (**a**) Correlations between the concentration of LBP and the percentage of Treg cells in the proximal small intestine, distal small intestine, and liver. (**b**) The correlation between the concentration of LBP and the percentage of Th17 cells in the proximal small intestine, distal small intestine, and caecum. (**c**) The correlation between the concentration of LBP and the percentage of Th1 cells in the proximal small intestine, distal small intestine, and liver. Spearman’s rank test was used.

To determine what kinds of changes are induced by BT, we used LBP as an indicator to analyse the correlation between the percentage of altered CD4^+^ T cells and the severity of BT in cirrhotic rats with BT. The percentage of Treg cells in the proximal small intestine was positively correlated with the serum concentration of LBP (ρ = 0.691, *P* < 0.05) (Fig. 5a), further validating the notion that BT causes an increase in the number of Treg cells in the proximal small intestine. The serum concentration of LBP was negatively associated with the percentage of Th17 cells in the proximal small intestine (ρ = −0.836, *P* < 0.01), the distal small intestine (ρ = −0.861, *P* < 0.01), and the caecum (ρ = −0.675, *P* < 0.05) (Fig. 5b), further supporting the hypothesis that BT causes a decrease in the number of Th17 cells in these tissues.

### CCl_4_-induced cirrhosis causes an increase in Tregs in the liver and a decrease in Th1 cells in the caecum and blood

First, the decrease in Th1 cells in the population of CD4^+^ T cells in the blood occurred only in the liver cirrhotic rats with BT, as mentioned above (Fig. 3f). Moreover, the percentage of Th1 cells in the CD4^+^ T cells in the blood of cirrhotic rats with and without antibiotics was significantly lower than the percentage in the normal control rats, but they was no significant difference between the cirrhotic rats with and without antibiotics (Fig. 4f), indicating that the decrease in Th1 cells in the blood of cirrhotic rats was a result of treatment with CCl_4_ and may be a possible cause of BT. Second, the percentage of Th1 cells in the CD4^+^ T cells in the caecum was lower in the cirrhotic rats with BT, without BT, with antibiotics and with placebo than in the normal controls, but there was no difference between the rats with BT, without BT, with antibiotics or without antibiotics and their respective normal controls (Fig. 4c). This indicates that the decrease in the number of Th1 cells in the caecum was the result of CCl_4_-induced cirrhosis and was not affected by BT. Third; the percentage of Tregs in the CD4^+^ T cells in the liver was higher in the cirrhotic rats with BT, without BT, with antibiotics or with placebo than in their respective normal controls. There was a significant difference between the cirrhotic rats with and without BT. This result indicates that the enrichment in Treg cells in the liver may have been caused in large part by the injection of CCl_4,_ and also by BT.

The percentage of Treg cells in the liver was positively correlated with the serum concentration of LBP (ρ = 0.760, P < 0.05) (Fig. 5a), indicating that Treg cells play an important role in the liver during the induction of BT. There was no significant correlation between the concentration of LBP and the percentage of Th1 cells in the caecum, further confirming that the changes in the number of Th1 cells in the caecum may not be affected by BT.

### BT may aggravate the cirrhosis caused decrease in Th1 cells in the proximal and distal small intestine and liver and increase in Th17 cells in the liver and the number of Tregs in the distal small intestine and colon

First, neither an increase in the percentage of Tregs in the distal small intestine and colon nor a decrease in the percentage of Th1 cells in the proximal and distal small intestine and liver, both of which appeared to be specific to cirrhotic rats with BT, as mentioned above, were observed between cirrhotic rats treated with antibiotics or placebo. This most likely indicates that BT contributed to the changes observed in these cells and does not exclude the notion that BT is an indicator of the different changes that were observed to be induced in these cells by CCl_4_. Second, the percentage of Th17 cells in the CD4^+^ T cell population in the liver was higher in the cirrhotic rats with BT, without BT, with antibiotics or with placebo than in their respective normal controls and also higher in the cirrhotic rats treated with placebo than in the cirrhotic rats treated with antibiotics, indicating that the injections of CCl_4_ were the main cause of the increase in Th17, while BT was a promotor of this increase.

The serum concentration of LBP was positively correlated with the percentage of Treg cells in the distal small intestine (ρ = 0.636, *P* < 0.05) (Fig. 5a), further indicating that BT plays an important role in the increase in Treg cells observed in the distal small intestine. The blood concentration of LBP was also negatively associated with the percentage of Th1 cells in the proximal small intestine (ρ = −0.809, *P* < 0.01), the distal small intestine (ρ = −0.802, *P* < 0.01) and the liver (ρ = −0.836, *P* < 0.05) (Fig. 5c), indicating that the Th1 cells in each of these sites are important for host defences against BT.

## Discussion

The characters and function of CD4^+^ T cells suggest that they potentially play important roles in the regulation of both BT and liver cirrhosis. On the one hand, CD4^+^ T cells are critical in the host to combat specific pathogens and to thereby prevent BT and infections, but their development and differentiation depend on the presence of and can adapt in response to microbes, which themselves affect liver cirrhosis^18^. On the other hand, liver diseases, such as liver cirrhosis, may influence liver and gut CD4^+^ T cells^19^,^20^, contributing to an imbalance in intestinal homeostasis and the development of BT. To the best of our knowledge, we are the first to disclose the presence of a major interaction between BT, CD4^+^ T cells and liver cirrhosis. We used a CCl_4_-induced rat model to perform this study, and we suggest that this model will be helpful for studies aimed at preventing the development of BT and liver cirrhosis.

We found that BT was positively associated with the severity of liver cirrhosis, and we suggest therefore that determining how to breaking the vicious circle between them would be of great clinical significance. Body weights were significantly lower and spleen weights and the volume of ascites were significantly higher in the cirrhotic rats with BT than in those without BT. The concentration of LBP, a marker of BT, was negatively associated with the levels of total protein and albumin but positively correlated with AST, TBil, and DBil levels. Cirrhotic rats treated with antibiotics had levels of liver damage that were similar to those observed in the cirrhotic rats without BT and liver function that was better than that observed in the cirrhotic rats treated with placebo (supplementary Fig. 1). Therefore, our results support the notion that intestinal decontamination with antibiotics or probiotics improves survival, liver function and complications in patients with cirrhosis^21^.

The mechanism by which BT affects liver cirrhosis is a subject of major concern. On the one hand, we found that there was an increase in the number of Tregs in the proximal small intestine and a decrease in the number of Th17 cells in the whole intestine and blood, and that these events are potentially caused by BT in cirrhotic rats. The enrichment of Treg cells observed in the gut may have been induced by bacterial components, such as capsular polysaccharide, or metabolites, such as short chain fatty acids, via BT^22^. Simultaneously, the reduction in bacteria in the intestine and the increase in bacteria in the extraintestinal organs that were induced by BT are considered to be major causes of the depletion observed in intestinal Th17 cells. In the absence of pathology, Th17 cells constitute a rare cell population; even though gut associated lymphoid tissue (GALT) is home to nearly 95% of the body’s CD4^+^ T-cells, including Th17 cells^23^. Pathogenic infections, such as those caused by extracellular bacteria, may result in a dramatic increase in the number of Th17 cells at the site of infection^24^. Th17 cells play important roles in the defence against microbes, particularly at mucosal sites, and in sustaining homeostasis in enterocytes. In contrast, Treg cells exert anti-inflammatory functions and control self-reactive T cells, including Th1, Th2, and Th17 cells. As a result, the enrichment of Treg cells and the depletion of Th17 cells in the intestine may cause further damage to intestinal barrier integrity and aggravate BT, thereby potentially leading to fatal complications, such as spontaneous bacterial peritonitis, in liver cirrhosis^25^. On the other hand, we observed that BT may aggravate the CCl_4_-induced decrease in Th1 cells in the proximal and distal small intestine and liver and increase in Th17 cells in the liver and Tregs in the distal small intestine and colon. Because Th1 cells are important for cytotoxic immunity to viruses and intracellular pathogens^26^ and Th17 cells are critical for the mucosal barrier defense against pathogens, BT may contribute to the weakened cellular but strengthened extracellular antimicrobial response in the liver and the proximal and distal small intestine. Because Th17 cells promote fibrosis whereas Th1 cells have an anti-fibrotic effect in the liver^27^, BT exacerbates liver cirrhosis. Hence, it may also be helpful for alleviating CCl_4_-mediated inflammation in the distal small intestine and colon, which can be accidentally induced as a result of its contribution to the upregulation of Treg cells.

Another important issue is the underlying cause of BT in liver cirrhosis. In this study, we found that only a decrease in Th1 cells in the blood of cirrhotic rats was largely induced by CCl_4_ and therefore a potential cause of BT. However, there was no significant correlation between the percentage of Th1 cells and the level of LBP in the blood of cirrhotic rats, indicating that even if the decrease in Th1 cells in the blood of cirrhotic rats contributed to BT, it was not a major factor. Consequently, alterations in CD4^+^ T cells were not observed to induce BT in rats with CCl4-induced liver cirrhosis. The destruction of components of the mucosal barrier, such as the mucus layer, the glycocalyx or a layer of epithelial cells, may be the first step to inducing BT during liver cirrhosis^28^.

In conclusion, we found that BT may cause an increase in the number of Treg cells in the proximal small intestine and a decrease in the number of Th17 cells in the whole intestine and blood in cirrhotic rats. It may also aggravate the CCl_4_-induced decrease in the number of Th1 cells in the proximal and distal small intestine, liver, caecum, and blood and the CCl4-induced increase in the number of Th17 cells in the liver and in the number of Tregs in the distal small intestine, colon, and liver. The results of this study may be helpful for further research into treatments for liver cirrhosis.

## Methods

All experimental procedures were conducted according to the 2011 National Institutes of Health Guide for the care and use of laboratory animals. The study was approved by the ethics committee of the First Affiliated Hospital, College of Medicine, Zhejiang University. All experimental animals received humane care.

### CCl_4_-induced liver cirrhosis

Cirrhosis was induced in male pathogen-free Sprague–Dawley rats (200–230 g initial weight) via the subcutaneous injection of a 50% (v/v) CCl_4_ solution in olive oil (Sigma-Aldrich, St. Louis, MO) into the dorsal region twice per week at a dose of 2 mL/kg. The dose was administered at the same time as 0.35 g/L Phenobarbital (Sigma-Aldrich) in the rats’ drinking water. In the control rats, CCl_4_ was replaced with olive oil. After 12–16 weeks, some of the rats had developed ascites, and in these cases, CCl_4_ and phenobarbital administration were continued for 2 weeks after the onset of ascites. The administration of phenobarbital and CCl_4_ was stopped 1 week before the experiments were performed.

### Study design

Out of the 80 rats that were treated with CCl_4_, 53 developed cirrhosis and ascites. This study consisted of 3 experiments. In protocol 1, we monitored the occurrence of BT in the intestinal bacteria in cirrhosis. In these rats, a total of 10^8^ RFP-marked *E. coli* were administered via gavage to 10 rats with cirrhosis and ascites and 6 healthy, phenobarbital-treated age- and sex-matched rats. In protocol 2, we determined the distribution of Treg, Th17 and Th1 cells in the entire intestinal lamina propria, liver and blood and evaluated their potential relationships with BT. In these experiments, we used 23 rats with cirrhosis and ascites in addition to 12 controls. In protocol 3, we investigated the effects of bowel bacterial decontamination on the distribution of Treg, Th17 and Th1 cells in 20 rats with cirrhosis and 16 control rats. To achieve this aim, after ascites onset, the animals were randomized in two groups that receive either broad-spectrum non-absorbable antibiotics, norfloxacin (10 mg/kg/day; Sigma-Aldrich) and vancomycin (16 mg/day; Sigma-Aldrich) or a placebo dissolved in drinking water. The treatments were administered orally for 2 weeks.

### Bacterial translocation assessment

A 10–15 mL volume of blood was obtained from each rat via puncture in the vena cava inferior. The MLNs of the ileocaecal area were aseptically isolated. After the isolates were ground, 100 μL of homogenized MLNs were cultured on MacConkey (Thermo Fisher Scientific, Waltham, MA), Mueller–Hinton (Thermo Fisher Scientific), and whole blood agar (Bio Merieux, Lyon, France) for 48 hours at 37 °C. BT was defined as the presence of viable organisms in the MLN culture^2^,^29^,^30^. To determine whether bacteraemia was present, 3 mL of blood was drawn from the inferior vena cava and inoculated into aerobic and anaerobic Bactec culture bottles. The cultures were incubated at 35 °C, and the growth value (a measurement of CO_2_ production by the bacteria) was continuously monitored for at least 7 days^4^. For BT monitoring, 10 cirrhotic rats and four control rats were lavaged with 10^8^ RFP-marked *E. coli.* The small intestine, colon, heart, lung, spleen, MLNs, kidneys, and liver were collected at 2 or 6 hours after lavage. The organs were rinsed in ice-cold PBS twice, and the RFP signal was visualised using a Clairvivo OPT Plus fluorescence microscope (Shimadzu Corporation, Kyoto, Japan) at a wavelength of 583 nm.

### Immune cell isolation

Peripheral blood mononuclear cells (PBMCs) were prepared from venous blood using density gradient centrifugation^31^. Intrahepatic lymphocytes were isolated as previously described^32^. Lamina propria lymphocytes (LPLs) were isolated from the proximal and distal small intestine, caecum, and colon as previously described^12^, with some modifications. Briefly, the intestine was removed and placed in ice-cold PBS supplemented with 5% heat-inactivated FCS. After the residual mesenteric fat was removed, the Peyer’s patches were carefully excised, and the intestine was opened longitudinally. The intestine was then thoroughly washed in ice-cold PBS and cut into 1.0-cm pieces. The pieces were incubated twice in 30 mL of 5 mM EDTA (Sigma-Aldrich) in RPMI media supplemented with 5% heat-inactivated FCS for 20 min at 37 °C with slow rotation. After each incubation, fresh EDTA (Sigma-Aldrich) was added, and the intraepithelial lymphocytes (IELs) in the epithelial cell layer were removed via filtration through a 100-mm cell strainer (BD Pharmingen, Franklin Lakes, USA). After the second EDTA incubation, the pieces were washed in PBS, cut into 1-mm^2^ pieces using a razor blade, and placed in 25 mL of digestion solution containing 5% FCS, 0.65 mg/mL collagenase VIII, and 1 unit/mL of DNase I. During digestion, the pieces were incubated at 37 °C for 20 min with slow rotation. After 20 min, the solution was strongly vortexed and then passed through a 100-μm filter (BD Pharmingen). The pieces were collected, and the digestion was repeated using fresh digestion solution. The supernatants were then combined, washed twice with 5% heat-inactivated FCS and suspended in 10 mL of a 40% fraction of Percoll gradient (General Electric Company, Fairfield, CT). The suspension was overlaid on 5 mL of the 80% fraction in a 15-mL Falcon tube (BD Pharmingen). Percoll gradient separation was performed using centrifugation for 20 min at 2500 rpm at room temperature. Lamina propria lymphocytes (LPLs) were collected in the interphase of the Percoll gradient, washed once, and resuspended in PBS supplemented with 0.3% (w/v) BSA and 0.09% (w/v) sodium azide. Isolated cells were used immediately for experiments.

### Surface and intracellular cytokine staining

Collected cells (1 × 10^6^ cells/mL) were suspended in staining buffer and stained for surface expression of CD3, CD4, and CD25. After surface staining, the cells were resuspended in Fixation/Permeabilization solution (BD Cytofix/Cytoperm kit, BD Pharmingen), and stained for intracellular Foxp3 expression according to the manufacturer’s protocol. To detect IFN- γ and IL-17A, lymphocytes (1 × 10^6^/mL) were stimulated for 5 hours with 2 μL Leukocyte Activation Cocktail with BD GolgiPlug™ (BD Pharmingen) in a cell culture incubator at 37 °C. The cells were stained for surface CD3 and CD4 expression and then fixed and permeabilized using a BD Cytofix/Cytoperm kit. The cells were then stained for intracellular IFN- γ and IL-17A. Cytokines were analysed using flow cytometry on a BD LSR II flow cytometer (BD Pharmingen), and the resulting data were analysed using FlowJo software (Treestar, Ashland, OR). The following antibodies were used: PE- or FITC-labelled anti-CD3 (G4.18, BD Pharmingen), PE- or FITC-labelled anti-CD25 (OX-39, BD Pharmingen), APC-labelled anti-CD4 (OX-35, BD Pharmingen), PerCP-labelled anti-CD8a (OX-8, BD Pharmingen), PE-Cyanine7-labelled anti-Foxp3 Ab (FJK-16s, eBioscience, San Diego, CA), PE-labelled anti-IFN- γ (DB-1, BD Pharmingen), and Alexa Fluor488-labelled anti-IL-17A (eBio17B7, eBioscience).

### Measurement of liver function

The serum concentrations of total protein (TP), albumin (Alb), globulin (Glb), alanine aminotransferase (ALT), aspartate transaminase (AST), alkaline phosphatase (ALP), and gamma-glutamyl transpeptidase (GGT) and total bilirubin (TBil), direct bilirubin (DBil), and indirect bilirubin (IBil) levels were measured using a standard clinical automated analyser (SRL, Tokyo, Japan).

### Lipopolysaccharide-binding protein ELISA

Blood samples were collected into endotoxin-free tubes (Endo Tube ET; Chromogenix AB, Sweden). After the samples were centrifuged, the blood plasma was stored at −80 °C. Plasma LBP levels were measured using an enzyme-linked immunosorbent assay (ELISA) kit (Cell Sciences, Newburyport, MA) according to the manufacturer’s instructions.

### Statistical analysis

The results are shown as the mean ± standard deviation. The normality of the distribution of the variables was evaluated using the Kolmogorov-Smirnov test. For multiple comparisons, differences among groups were determined using one-way ANOVA and the unpaired Student’s t test with Bonferroni’s correction. Spearman’s rank correlation was used to determine correlations between variables. Statistical significance was set at 0.05. All statistical analyses were performed using SPSS 19.0 for Windows (SPSS, Inc., Chicago, USA).

## Additional Information

**How to cite this article**: Shi, H. *et al*. Bacterial translocation aggravates CCl_4_-induced liver cirrhosis by regulating CD4^+^ T cells in rats. *Sci. Rep.* **6**, 40516; doi: 10.1038/srep40516 (2016).

**Publisher's note:** Springer Nature remains neutral with regard to jurisdictional claims in published maps and institutional affiliations.

## Supplementary Material

Supplementary Figure 1

## Figures and Tables

**Table 1:** Characteristics of normal rats and cirrhotic rats with or without BT.

Indexes	Normal	Without BT	BT
	(n = 12)	(n = 12)	(n = 11)
Total protein (g/dL)	5.1 ± 0.3	5.0 ± 1.3	4.1 ± 0.6*^,#^
Albumin (g/dL)	3.3 ± 0.2	2.6 ± 0.9**	1.5 ± 0.4*^,#^
Globulin (g/dL)	1.8 ± 1.5	2.5 ± 0.2**	2.5 ± 0.1*
Albumin/Globulin	1.9 ± 0.1	1.0 ± 0.3**	0.7 ± 0.2*^,#^
ALT (U/L)	49.6 ± 13.9	154.6 ± 34.6	228.3 ± 159.0*
AST (U/L)	76.5 ± 11.2	318.9 ± 94.6	685.2 ± 670.8*
ALP (U/L)	29 ± 32.6	165.7 ± 110.0**	198.9 ± 113.4*
TBIL (μmol/L)	1.8 ± 0.6	21.7 ± 15.1**	27.7 ± 19.5*
DBIL (μmol/L)	1.8 ± 0.6	18.9 ± 12.3**	24.0 ± 16.3*
IBIL (μmol/L)	0 ± 0.9	2.9 ± 3.0	3.7 ± 3.4*
GGT (U/L)	−2.9 + 1.4	16.3 ± 10**	16.6 ± 14.6*
Initial body weight (g)	212.5 ± 8.1	213.0 ± 5.6	213.6 ± 7.0
Final body weight (g)	469.5 ± 11.3	379.5 ± 17.1**	346.2 ± 50.6*^,#^
Body weight increase (g)	257.0 ± 10.1	166.6 ± 16.0**	132.6 ± 46.7*^,#^
Spleen weight (g)	0.9 ± 0.1	1.4 ± 0.3**	1.7 ± 0.4*^,#^
Spleen weight/Final body weight (%)	0.19	0.36	0.49
Ascites amount (mL)	/	16.2 ± 7.5	25.7 ± 5.4^#^
Ascites/Final body weight (mL/kg)	/	43.1 ± 20.9	76.0 ± 21.0^#^

Abbreviations: ALT, alanine aminotransferase; AST, aspartate aminotransferase; ALP, alkaline phosphatase; GGT, gamma-glutamyl transpeptidase; TBIL, total bilirubin; DBIL, direct bilirubin; IBIL, indirect bilirubin; GGT, γ-glutamyl transpeptidase. The data are presented as the means ± SD. *P < 0.05, BT versus Normal; **P < 0.05, Without BT versus Normal; ^#^P < 0.05, BT versus Without BT, one-way ANOVA.

**Table 2:** Characteristics of Placebo- and Antibiotic-Treated Cirrhotic and Control Rats.

Indexes	Cirrhotic Rats		Control Rats	
	Placebo (n = 10)	Antibiotics (n = 10)	Placebo (n = 8)	Antibiotics (n = 8)
Total protein (g/dL)	4.2 ± 0.8	5.0 ± 0.8	5.0 ± 0.4	5.2 ± 0.2
Albumin (g/dL)	1.9 ± 1.0	3.0 ± 0.3^#^	3.3 ± 0.2*	3.4 ± 0.2
Globulin (g/dL)	2.6 ± 0.6	2.4 ± 0.3	1.8 ± 0.1*	1.8 ± 0.2**
Albumin/Globulin	0.7 ± 0.3	1.3 ± 0.3^#^	1.9 ± 0.1*	1.9 ± 0.2**
ALT (U/L)	204.5 ± 94.8	112.2 ± 24.6^#^	44.0 ± 14.9*	56.4 ± 10.2**
AST (U/L)	546.3 ± 364.5	265.7 ± 56.9^#^	70.7 ± 9.7*	83.6 ± 9.0**
ALP (U/L)	221.1 ± 132.3	109.5 ± 53.8	28.3 ± 36.8*	30.0 ± 31.1**
GGT (U/L)	24.2 ± 13.4	16.2 ± 8.6	−2.7 + 1.6*	−3.2 + 1.1**
TBIL (μmol/L)	39.3 ± 16.4	20.8 ± 10.7^#^	2.0 ± 0.0*	1.6 ± 0.9**
DBIL (μmol/L)	33.7 ± 13.9	16.5 ± 7.1^#^	2.0 ± 1.3*	2.4 ± 0.9**
IBIL (μmol/L)	5.2 ± 3.0	2.0 ± 2.6	−0.3 + 1.5*	−0.8 + 1.1
Initial body weight (g)	217.2 ± 4.4	213.8 ± 7.3	214.2 ± 8.6	210.8 ± 8.0
Final body weight (g)	377.7 ± 48.5	390.0 ± 16.8	469.8 ± 10.2*	469.2 ± 13.3**
Body weight increase (g)	160.5 ± 45.7	176.2 ± 15.0	255.7 ± 11.4*	258.3 ± 9.4**
Spleen weight (g)	1.6 ± 0.3	1.3 ± 0.3^#^	0.9 ± 0.1*	0.9 ± 0.0**
Spleen weight/Final body weight (%)	4.4 ± 1.0	3.2 ± 0.6^#^	1.8 ± 0.1*	1.9 ± 0.1**
Ascites amount (mL)	24.3 ± 3.4	8.5 ± 2.3^#^	/	/
Ascites/Final body weight (mL/kg)	64.6 ± 7.2	23.4 ± 6.0^#^	/	/

Abbreviations: ALT, alanine aminotransferase; AST, aspartate aminotransferase; ALP, alkaline phosphatase; GGT, gamma-glutamyl transpeptidase; TBIL, total bilirubin; DBIL, direct bilirubin; IBIL, indirect bilirubin. The data are presented as the means ± standard deviation. *P < 0.05, placebo-treated normal rats versus placebo-treated cirrhotic rats; **P < 0.05, antibiotics-treated normal rats versus antibiotics-treated cirrhotic rats; ^#^P < 0.05, antibiotics- versus placebo-treated cirrhotic rats. The unpaired Student’s t test with Bonferroni’s correction was used.
