# Supplementary material for: Bacterial translocation aggravates CCl4-induced liver cirrhosis by regulating CD4+ T cells in rats
Source: Sci Rep. 2017 Jan 30;7:40516. doi: 10.1038/srep40516 (PMC5278361; doi:10.1038/srep40516)
Supplement: Supplementary Figure 1 [file srep40516-s1.pdf]

Bacterial translocation aggravates CCl_4-induced liver cirrhosis by regulating $CD4^+$ T cells in rats

Haiyan Shi[1,2#], Longxian Lv[1,2#], Hongcui Cao[1,2], Haifeng Lu[1,2], Ning Zhou[1,2], Jiezuan Yang[1,2], Haiyin Jiang[1,2], Huihui Dong, Xinjun Hu[1,2], Wei Yu[1,2], Xiawei Jiang[1,2], Beiwen Zheng[1,2], Lanjuan Li[1,2]*

Supplementary figure 1

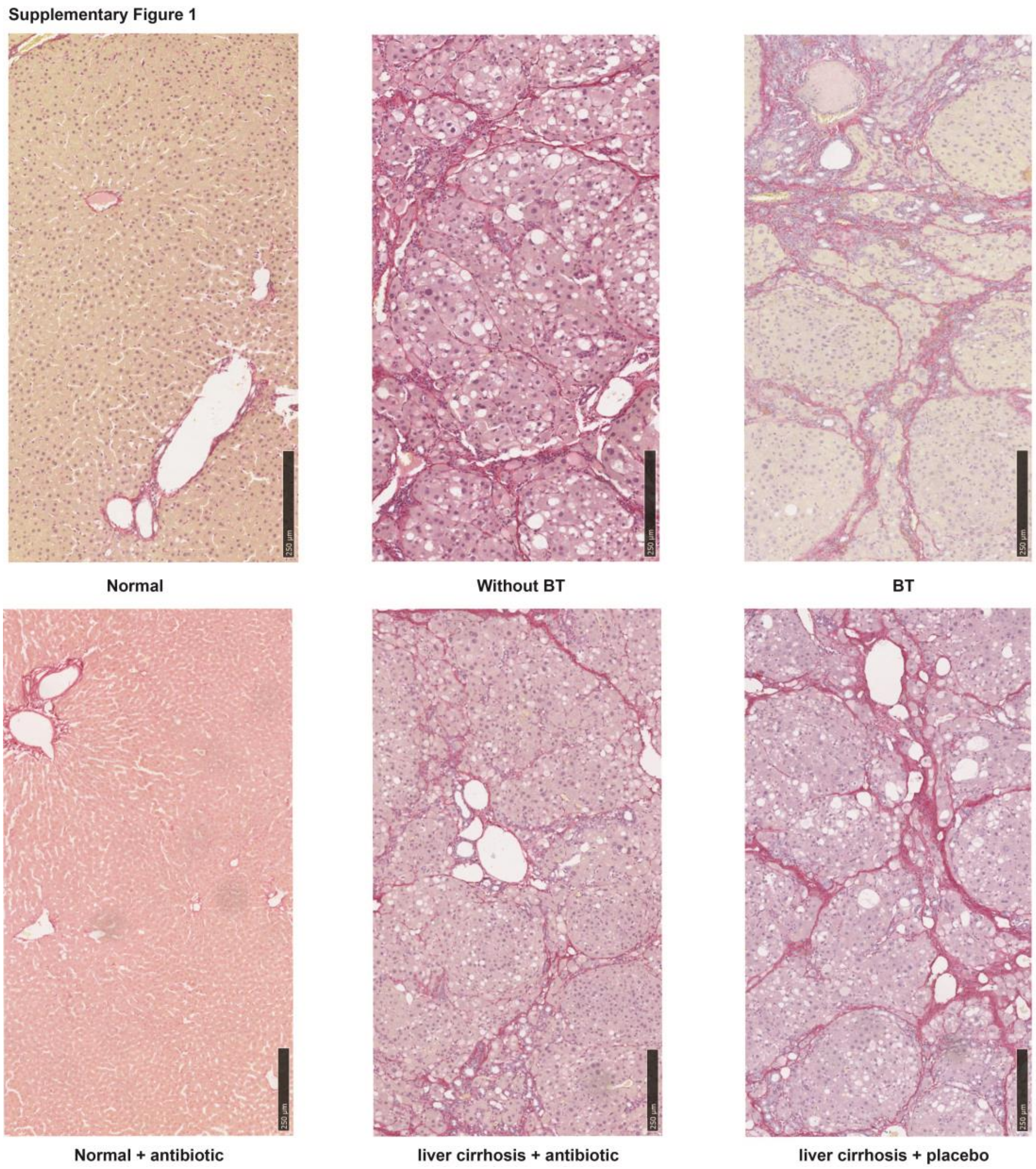

Supplementary Figure 1. BT aggravates the **CCl_4-** induced liver injury. To visualize collagen deposition, the formalin-fixed, paraffin-embedded liver samples were stained with Sirius Red. Histological examinations and representative images were taken using a NanoZoomer 2.0 RS optical microscope.
